# Genetic data suggest gene flow within a narrow hybrid zone between two recently separated species in the genus *Parnassius* (Lepidoptera: Papilionidae)

**DOI:** 10.1371/journal.pone.0321742

**Published:** 2025-04-24

**Authors:** Paolo Gratton, Valentina Todisco, Donatella Cesaroni, Valerio Sbordoni, Vazrick Nazari

**Affiliations:** 1 Department of Biology, University of Tor Vergata, Roma, Italy; 2 Department of Environment and Biodiversity, Paris Lodron University of Salzburg, Salzburg, Austria; 3 Department of Biology, University of Padova, Padova, Italy; Sunrise University, INDIA

## Abstract

Length polymorphism analysis of six microsatellite loci for 540 Clouded Apollo butterflies from Italy and Switzerland revealed a clear separation between sister species *Parnassius mnemosyne* and *P. turatii* and provided updated limits for their respective ranges*.* Correspondence analysis and Structure clustering of a subset of 140 individuals from the Eastern Alps determined intermediate genetic makeup for three small samples collected in the mid Piave valley (northern Italy). The genotypes of the intermediate individuals are not consistent with F1 hybrids, hinting at clinal genetic variation. Our data indicate a narrow introgression zone with a shallow depth of 50–100 km in what is likely to be the only area of contact between the proposed species *P. mnemosyne* and *P. turatii*. Our findings indicate incomplete reproductive isolation between the two species, and are consistent with selection against hybrids or with a recent establishment of a secondary contact. The latter may result from slow recolonization of the Eastern Alps from glacial refugia or from very recent changes in traditional land management practices such as grazing and mowing at semi-natural grasslands.

## Introduction

**Fig 1:**
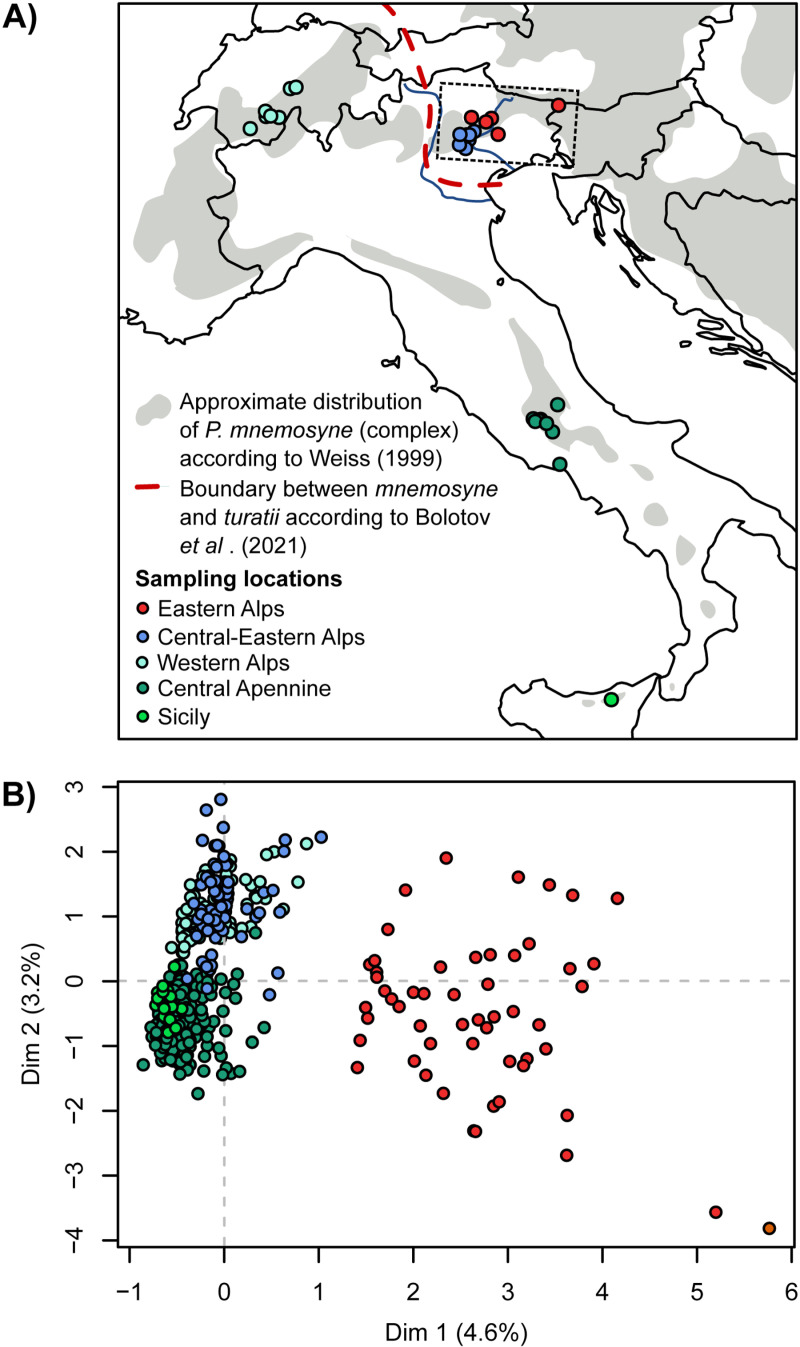
A) Map of sampling locations for this study, also showing the distribution of the *P. mnemosyn*e complex and the boundary between the ranges of *P. mnemosyne* and *P. turatii* proposed by Bolotov et al. [ 8]. The dashed rectangle indicates the area enlarged in Fig 3A. Continuous lines represent country borders. B) Correspondence analysis on multilocus genotypes at six microsatellite loci for 540 individuals of the *P. mnemosyne* complex. Dots color indicates geographic region as in Fig 1A. Note that the subdivision between Central-Eastern and Eastern Alps is based on genetic structure rather than geography alone. Color codes for these two regions have been selected to match those employed in Figs 2 and 3 for ease of comparison. The map was created using Natural Earth (www.naturalearthdata.com, in Public Domain).

Despite recent hostility expressed towards usage of the term “cryptic species” in biodiversity studies [1], cryptic species remain very real and well defined, at least in entomology, as species that have traditionally escaped detection due to external similarity to a sister species, and that are usually discovered through genetic or molecular techniques [2–5]. One such case concerns recent revelations that the well-known clouded Apollo butterfly (*Parnassius mnemosyne* Linnaeus, 1758) is in fact a complex of very similar species that occupy different geographic ranges across Eurasia [6–9]. However, the exact nature of the relationship between these species and the degree (or lack) of gene flow between them has so far remained unstudied. Butterflies in the genus *Parnassius*, characterized by patchy distributions in the northern hemisphere and limited dispersal ability, are a popular model in phylogenetic, metapopulation, population genetics and gene flow studies (*e.g.*, [10–16]). In the last decades, phylogeographic studies [6,7] have revealed significant genetic differentiation among populations of the clouded Apollo butterflies, all of which were until then attributed to the same species, *Parnassius mnemosyne*. In particular, a deep divergence in mitochondrial DNA was observed between the westernmost populations (Pyrenees, Sicily, peninsular Italy, western Alps) and all other populations across the range of the species (see Fig 1), which includes most of Europe and western-central Asia. The remarkable degree of sequence divergence in the *cox1* gene (*ca.* 4%) between the two groups immediately suggested that this could represent a case of cryptic speciation [6,8]. Further evidence in this direction came from nuclear markers (*EF*-1*a* and microsatellite DNA) [6]. This pattern, which is also observed in other European butterfly species (*e.g.*, [17]), is typically interpreted as the result of allopatric divergence in different Pleistocene glacial refugia, with divergent lineages that may then establish hybrid zones where secondary contact occurred. However, in the case of the *P. mnemosyne* complex, lack of adequate sampling did not yet allow to establish whether and where secondary contact actually occurred between the two groups and estimate their degree of reproductive isolation. Nonetheless, Bolotov et al. [8] considered the available data sufficient to propose the separation of the western populations from *P. mnemosyne* sensu stricto as a distinct species, *P. turatii* Fruhstorfer, 1908 (see [18,19] for discussion on the nomenclatural unavailability of the originally proposed name *P. nebrodensis* Turati, 1907).

Bolotov et al. [8] did not find clear diagnostic characters in the wing venation, but recognized “clear differences” in the male genitalia structure between *P. mnemosyne* from Moldova and Belarus and *P. turatii* from France, suggesting that these differences may represent an effective mechanism of prezygotic reproductive isolation. The very large geographic distance separating those samples, however, does not allow to precisely gauge the relevance of this finding in terms of evidence for an isolating mechanism. The two species also consume different host plants in some parts of their distribution, with *P. turatii*’s larvae feeding only on *Corydalis solida* in Sicily and the Pyrenees, while larvae of *P. mnemosyne* are known to feed on four species of *Corydalis* (*C. solida*, *C. intermedia*, *C. cava* and *C. pumila*). However, these differences appear to be simply driven by the availability of potential food plants in different geographic areas (see [20]).

On the other hand, the exact geographical boundaries of the two species have remained poorly defined: Bolotov et al. [8] suggested the Adige valley as a probable eastern limit for *P. turatii* (Fig 1A), while the westernmost individuals attributable to *P. mnemosyne sensu stricto* come from the Cansiglio woods, about 100 km east of the Adige [8]. In reality, the data available to Bolotov and collaborators did not allow to establish whether the two species actually occur in sympatry or come into secondary contact with potential gene exchange. Here we analyse new microsatellite DNA polymorphism data from populations of *P. mnemosyne* and *P. turatii* from the Alps, Italian peninsula and Sicily to determine the range of the two species and assess genetic introgression between them.

## Materials and methods

We analyzed length polymorphism at six microsatellite loci (mnemoB2, mnemo30, mnemo96, mnemo100, mnemo111, mnemo172) from a total of 540 individual butterflies from 28 locations in Italy and Switzerland (Fig 1A, S1 Table). A subset of these data have already been presented by Gratton [6]. DNA extraction, amplification and genotyping procedures are described by Gratton [6] and Gratton & Sbordoni [21].

We explored genetic structure by fitting correspondence analyses (CA) on microsatellite genotype data using the function *ca* from the “ca” R package [22], after converting genotype data into a *genind* object using function *df2genind* from the “adegenet” R package [23]. We started by applying correspondence analysis to the full data set, and later focused on subsets of data defined according to geography and genetic clustering. In particular, we focused on genotypic profiles of 102 individuals collected in the Eastern Alps, where the two genetic clusters attributable to *P. mnemosyne* and *P. turatii* are found in close geographic proximity. This subset of data was also analysed by running the Bayesian clustering algorithm implemented in Structure 2.3.4 [24]. We ran five independent MCMC with 1 million generations each, setting the number of clusters to *K* = 2 to represent the expectation of two gene pools corresponding to the two proposed species. We did not use geographic location as clustering prior (USEPOPINFO = 0). We compared the results of CA and Structure between them and to the previously proposed distribution of *P. turatii* and *P. mnemosyne*, based on mtDNA sequences to validate that CA’s first dimension could be used to derive an index of genetic attribution to each of the two putative taxa. We then plotted the average value of this index at each sampling location in the geographic space to visualize the location and steepness of the geographic gradient between *P. turatii* and *P. mnemosyne*. All plots were generated in R 4.2.0 [25].

We initially observed that three of the employed microsatellite loci (*mnemoB2*, *mnemo30*, and *mnemo96*) showed distinct geographic patterns in the frequency of missing data (S1 and S2 Figs) which appear to be concentrated in different areas at each locus. Remarkably, none of these areas were located in peninsular Italy, where primers for microsatellite loci had been developed [21] (S1 Fig). This pattern strongly suggests the existence of null alleles at these loci. Based on the frequency of missing data (S1 and S2 Figs), null alleles may have a frequency of as much as *ca.* 80% in some populations (*e.g.*, locus m30 in central-eastern Alps, see S1 and S2 Figs). However, the geographic clustering of missing data hints at the presence of null alleles with a common evolutionary origin, which can be, therefore, confidently interpreted as a single genetic lineage. We used the RECESSIVEALLELES = 1 setting to allow the Structure software to correctly interpret the data from genetic loci containing null alleles, and we fitted Correspondence Analyses by imputing missing data in the aforementioned loci as homozygous genotypes for a “ghost” allele.

We checked for patterns of Isolation by Distance (IBD) by computing pairwise Weir and Cockerham’s *F*_ST_ values among sampling locations using the *pairwise.WCfst* function from the ‘hierfststat’ R package [26] and fitting separate linear regressions of *F*_ST_ versus the logarithm of geographic distance (computed by the function *distHaversine* from the ‘geosphere’ R package, [27]) for pairs of locations clearly attributed to the same species, to different species or involving samples that appeared to have mixed genetic makeup in previous analyses (S3 Fig).

## Results

The correspondence analysis (CA) on the whole data set separates, along the first dimension, all individuals collected in the easternmost Alpine locations from the remaining samples from Sicily, central Apennines, Switzerland and central-eastern Alps (Fig 1B). This clustering largely matches the supposed distribution of *P. mnemosyne* (the eastern cluster) and *P. turatii* (the western cluster).

**Fig 2:**
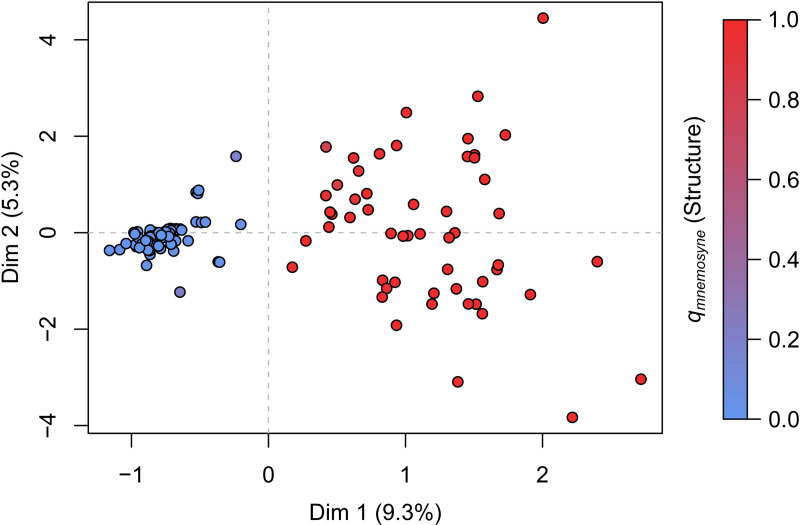
Correspondence analysis on multilocus genotypes at six microsatellite loci for 140 individuals of the *P. mnemosyne* complex sampled in Central/Eastern Alps (see Figs 1A and 3A). Dots color indicates ancestry coefficient *q* in the eastern genetic cluster (*mnemosyne*) computed by the Structure software.

**Fig 3:**
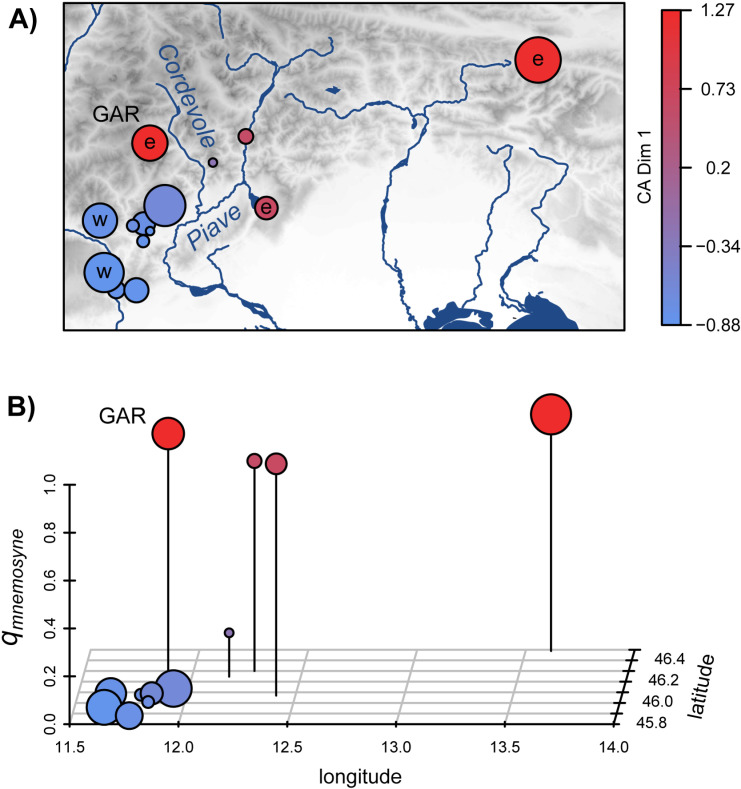
A) Map of central/eastern Italian Alps showing the sampling locations for 140 individuals of the *P. mnemosyne* complex. Letters show locations with available mtDNA sequences [7,28] (“w” indicates the western mitochondrial clade and “e” the eastern clade). B) Mean value of the ancestry coefficient *q* in the eastern (*mnemosyne*) genetic cluster from Structure analysis for each sampling location plotted against its longitude and latitude. In both A and B, dot colors indicate the mean value of the Dimension 1 of the correspondence analysis (CA). The position of the outlier Val di Gares (GAR) sample (see Discussion section) is highlighted in both panels. Map created using Natural Earth (www.naturalearthdata.com); in Public Domain.

The CA fitted on the subset of data from the Alps east of the Adige Valley showed a similar separation along the first dimension of individuals from the easternmost Alpine locations Fig 2). Results of the Structure clustering with *K* = 2 on the same subset strictly matched CA, with an *R*^2^ of 0.91 between CA dimension 1 and the *logit* transformation of Structure’s ancestry coefficient *q* (see Fig 2). Both CA dimension 1 and Structure’s *q* may be, thus, taken to represent a *mnemosyne* - *turatii* genetic index. The plot of average values of the ancestry coefficient *q* in the eastern (‘*mnemosyne*’) genetic cluster (*q*_*mnemosyne*_) at each location on the geographic space (Fig 3) shows that most locations display extreme values, which are low to the west (*turatii*) and high to the east (*mnemosyne*). Three samples, however, are characterized by somewhat intermediate values, and all of them were collected in geographically intermediate locations between strongly *turatii* and strongly *mnemosyne* samples, a pattern suggestive of some level of clinal introgression (Fig 3). The intermediate average values of *q*_*mnemosyne*_ at these locations did not result from a mixture of ‘pure’ individuals, but from intermediate *q* values assigned to most individuals (S2B Fig).

Interestingly, none of the investigated microsatellite loci was fixed or nearly fixed for alternative alleles in the two ‘forms’, but strong differences in allele frequencies along a West to East direction in the Eastern Alps were obvious at all loci. A few alleles appeared to be ‘typical’ of either *turatii* or *mnemosyne* (*i.e.*, they were common in locations unambiguously assigned to one form and absent or extremely rare in the other, S2A Fig). Locations in the potential ‘hybrid’ zone contain the typically *turatii* allele m100.97 and the typically *mnemosyne* alleles m30.234, m30.196, m172.112 and B2.104 (see S2A Fig). However, there are no such alleles that occur in heterozygosity in the potential ‘hybrid’ zone. While our data are insufficient for a precise determination of ancestry, the latter observation strongly suggests that intermediate individuals do not represent F1 hybrids.

The linear regression of genetic differentiation (*F*_ST_) on geographic distance (S3 Fig) for locations that were clearly attributed to different ‘species’ did not show any increase of differentiation with geographic distance. Conversely, a pattern of isolation by distance, whereby genetic differentiation increases with geographic distance, was obvious in the within ‘species’ comparisons. Interestingly, comparisons involving the four samples in the potential ‘hybrid’ zone show a similar trend of increasing genetic differentiation with increasing geographic distance but with a raised intercept, consistent with their mixed genetic makeup as evidenced in CA and Structure analysis.

## Discussion

Hybridization is not rare in butterflies, and any slight overlap in morphology, behaviour and ecology are likely to allow it to occur [29,30]. Hybridization is an important generator of genetic diversity and its impact on the process of speciation is well documented: It may slow down or reverse differentiation by allowing gene flow and recombination, or alternatively, it may accelerate speciation via adaptive introgression or hybrid speciation (e.g., [31]).

Our analysis of butterflies in the *P. mnemosyne* complex show that the genetic features of a few geographically intermediate localities in the mid Piave Valley are themselves intermediate between typical *turatii* and typical *mnemosyne* populations. In fact, with the exception of the GAR sample (Val di Gares, Gares Valley), our Fig 3 indicates a clinal trend of genetic variation, albeit rather steep, suggestive of genetic introgression between the populations attributed to the two putative species. Despite the small number of genetic markers that we have analyzed, these samples show a mixture of typically ‘mnemosyne’ alleles at some loci and typically ‘turatii’ alleles at others. Patterns of isolation by distance (IBD) also indicate these local populations as somewhat intermediate, in that their differentiation with respect to samples from other locations increases with geographic distance, but they appear more genetically differentiated for the same geographic distance than properly ‘conspecific’ populations (S3 Fig). The outlier position of the Gares valley sample may be interpreted in the light of its geographic location and local topography. The valley is surrounded by high mountains (> 2000 m) and opens downstream into the upper valley of the Cordevole creek, the main tributary of the Piave river (Fig 3). The conformation of the territory, therefore, likely isolates the GAR population from the other sampled populations in the mid Piave valley. This may explain the outlier position of the GAR sample with respect to the apparent clinal transition between *P. turatii* and *P. mnemosyne* genetic clusters observed in the mid Piave valley. Our analyses, however, rely on a rather sparse sampling of the apparently genetically intermediate region, and on a very small set of highly variable microsatellite DNA markers. A more detailed investigation of this interesting system would require more dense geographic and genomic sampling, allowing for a proper statistical analysis of clinal differentiation in this highly anisotropic geographic space.

Nonetheless, within their limits, our results clearly hint at the existence of some gene flow between the two species, suggesting an introgression zone with a “depth” of *ca.* 50–100 km exists between *P. turatii* and *P. mnemosyne*. The limited depth of the introgression zone is consistent with significant *post-copula* reproductive isolation, possibly due to selection against hybrids. Importantly, as far as it is known, the mid Piave valley represents the only geographic corridor continuously inhabited by populations of *P. mnemosyne* or *P. turatii* (see Fig 1A, range map by Weiss [32], reprised by Bolotov et al. [8] and GBIF data obtained from https://www.gbif.org) and thus, it is likely the only region where contact between these two lineages actually occurs. This distribution matches the rarity or absence of these butterflies’ larval food plants (*Corydalis* sp.) in the Upper Adige valley ([20] and GBIF data^1^). In the mid Piave valley, the presence of these butterflies is limited to the north by the Belluno Dolomites, which represent an important obstacle for their dispersal, and to the south by the Venetian plain, where there are no suitable habitats. The “width” of the potential hybrid zone is therefore probably less than 30 km. Past authors who have studied this biological system (e.g., [6,8]) have considered that the differentiation between *P. turatii* and *P. mnemosyne* is primarily due to evolution in allopatric isolation, likely into separate Pleistocene glacial refugia. The hybrid zone whose existence is suggested by our data should therefore be considered the result of a secondary contact. Since the mid Piave valley was occupied by glaciers (certainly unsuitable environment for these butterflies) until about 15 ka BP, the secondary contact between the two species certainly dates back to not earlier than this date. In fact, we have no actual information to put a lower limit to the age of the secondary contact, which could even be much more recent if post-glacial colonization of central-eastern Alps happened at a relatively slow pace. However, historical changes in land management by humans, including the recent cessation of traditional management practices such as grazing and mowing at semi-natural grasslands and coppicing in woodlands may also have altered, and, at least at times, increased, habitat connectivity, possibly favoring contact between the two lineages recognised as *mnemosyne* and *turatii* [33]. The hybridization between *mnemosyne* and *turatii* might be an ongoing evolutionary process that facilitates adaptation and persistence in a rapidly changing world. It may drive the emergence of new biodiversity representing priorities for conservation programmes. This hypothesis remains, however, highly speculative and more dense genomic and geographic sampling will be needed for a more detailed investigation of this fascinating biological system.

## Supporting information

S1 Fig Proportion of individuals with missing data at each locus and sampling location. Darker colors indicate higher proportions (see legend). Locations appear in an approximately north-east to south-west order, as in Appendix 1, highlighting the geographic clustering of missingness at loci B2, m30 and m96.
(PDF)

S2 Fig A) Graphical representation of the microsatellite genotypes of 140 individuals sampled in Central and Eastern Alps. Color codes for genotypes are provided in the internal legend (topright). No locus is fixed or nearly fixed for alternative alleles in the two forms, but strong differences in allele frequencies are obvious at all loci. Alleles that are ‘typical’ of either *turatii* or *mnemosyne* (i.e., they are common in locations unambiguously assigned to one form and absent or extremely rare in the other) are highlighted in blue (for *turatii*) or red (for *mnemosyne*). Locations in the potential ‘hybrid’ zone contain the typically *turatii* allele m100.97 and the typically *mnemosyne* alleles m30.234, m30.196, m172.112 and B2.104. **B) Full Structure barplot.** Dots indicate the point estimate of ancestry in the western ‘turatii’ genetic cluster (*q*_*mnemosyne*_) for each individual. Individuals are grouped by location. Blue areas represent the average *q*_*mnemosyne*_ at each location, while red areas represent 1-*q*_*mnemosyne*_ (i.e., the average fraction assigned to the ‘*turatii*’ cluster). **C) Map of sampling locations.** Circle areas are proportional to sample size and color scaled according to average ancestry parameter (*q*_*mnemosyne*_).
(PDF)

S3 Fig Isolation by distance. Plot of FST vs. great circle geographic distances (in log scale) between all pairs of locations in the complete dataset. Thick lines represent linear regressions and shaded areas their 95% CIs. The regression for between ‘species’ comparisons is nearly flat, while a pattern of isolation by distance is obvious in the within ‘species’ comparisons. Interestingly, comparisons involving the four samples in the potential ‘hybrid’ zone (BCH, TVN, LAP, CAN, see S2 Fig) show a similar trend of increasing genetic differentiation with increasing geographic distance but with a raised intercept, consistent with their mixed genetic makeup as evidenced in CA and Structure analysis.
(PDF)

S1 Table Samples employed in this study (n = number of genotyped individuals). (DOCX)
