# Supplementary figures and images for: Genetic data suggest gene flow within a narrow hybrid zone between two recently separated species in the genus Parnassius (Lepidoptera: Papilionidae)
Source: PLoS One. 2025 Apr 24;20(4):e0321742. doi: 10.1371/journal.pone.0321742 (PMC12021245; doi:10.1371/journal.pone.0321742)

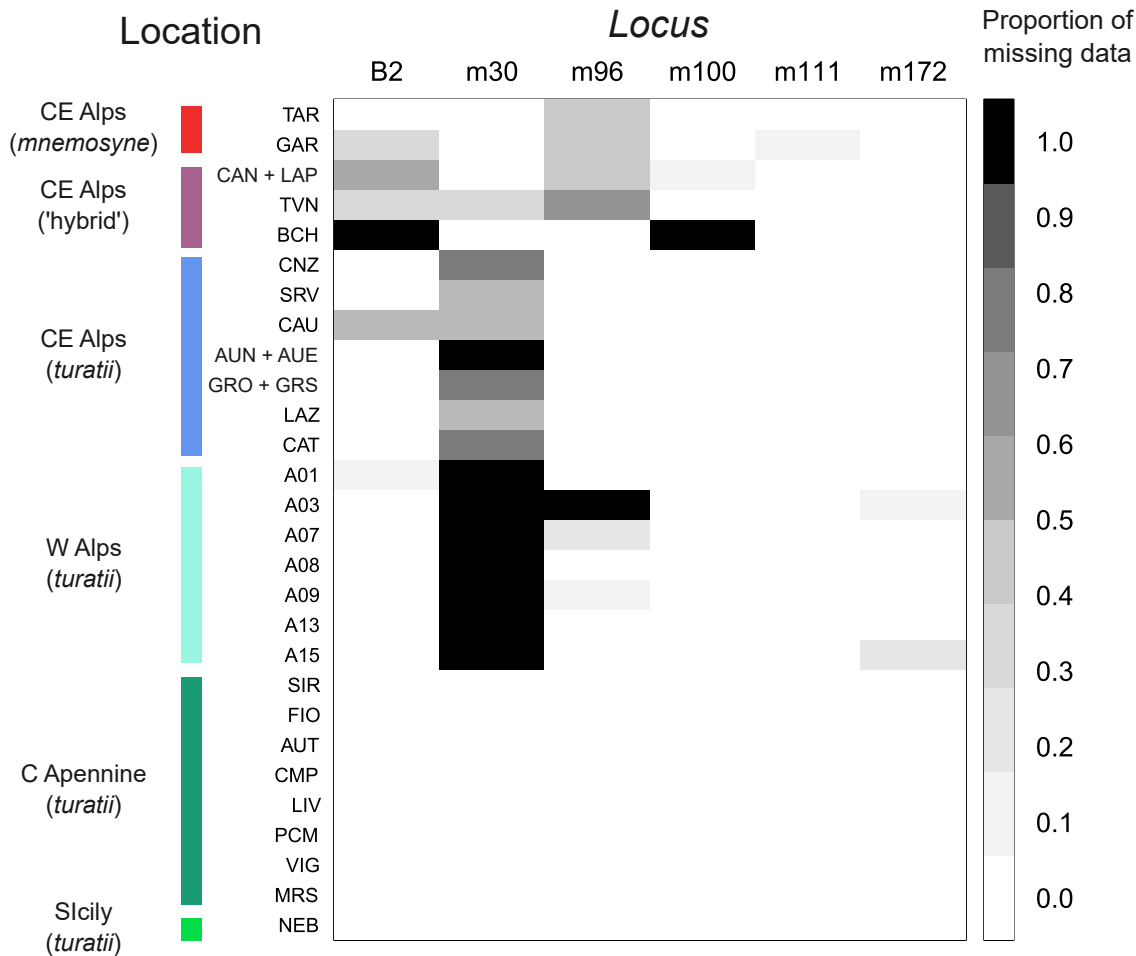

Supplement: S1 Fig — Darker colors indicate higher proportions (see legend). Locations appear in an approximately north-east to south-west order, as in Appendix 1, highlighting the geographic clustering of missingness at loci B2, m30 and m96. (PDF) [file pone.0321742.s001.pdf]

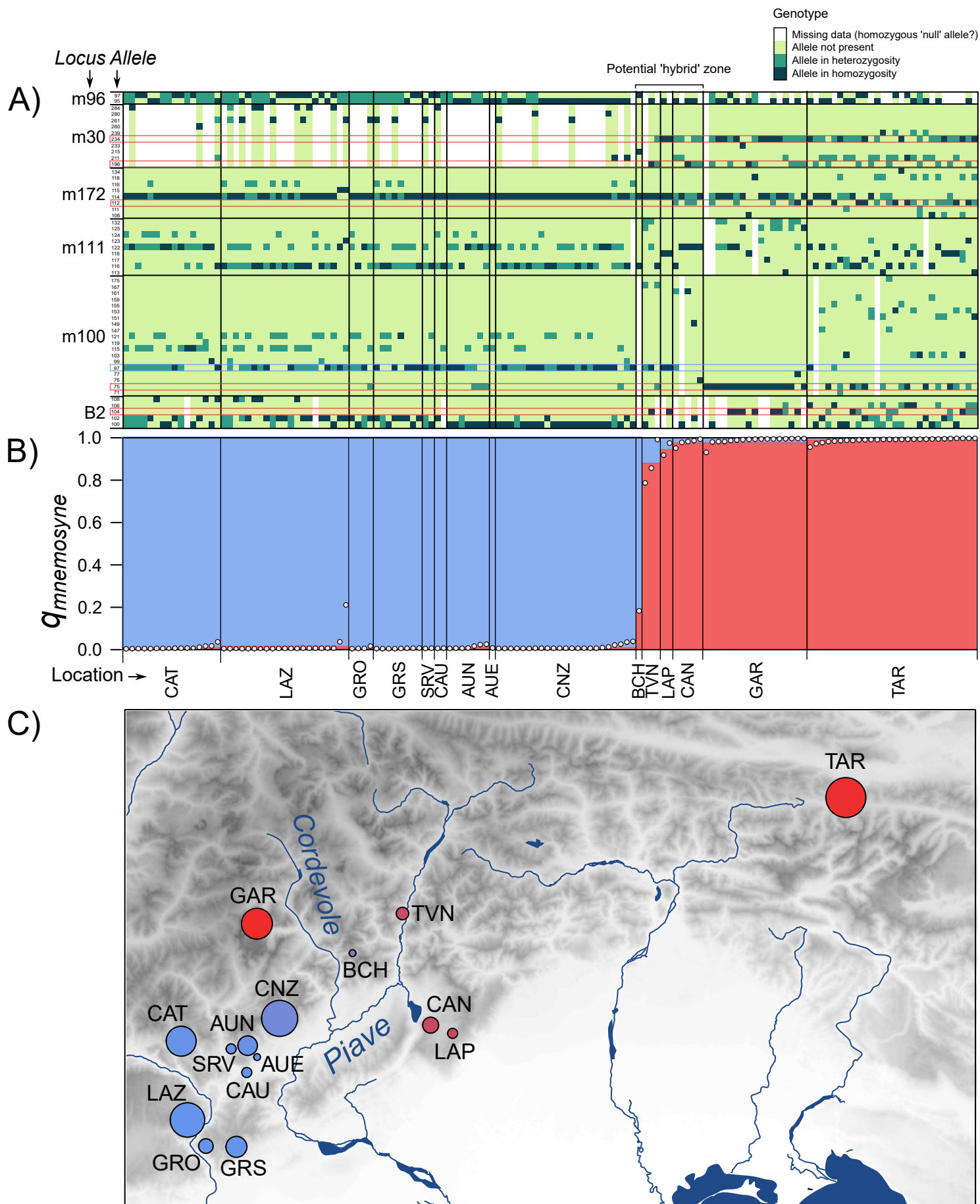

Supplement: S2 Fig — Color codes for genotypes are provided in the internal legend (topright). No locus is fixed or nearly fixed for alternative alleles in the two forms, but strong differences in allele frequencies are obvious at all loci. Alleles that are ‘typical’ of either turatii or mnemosyne (i.e., they are common in locations unambiguously assigned to one form and absent or extremely rare in the other) are highlighted in blue (for turatii) or red (for mnemosyne). Locations in the potential ‘hybrid’ zone contain the typically turatii allele m100.97 and the typically mnemosyne alleles m30.234, m30.196, m172.112 and B2.104. B) Full Structure barplot. Dots indicate the point estimate of ancestry in the western ‘turatii’ genetic cluster (qmnemosyne) for each individual. Individuals are grouped by location. Blue areas represent the average qmnemosyne at each location, while red areas represent 1-qmnemosyne (i.e., the average fraction assigned to the ‘turatii’ cluster). C) Map of sampling locations. Circle areas are proportional to sample size and color scaled according to average ancestry parameter (qmnemosyne). (PDF) [file pone.0321742.s002.pdf]

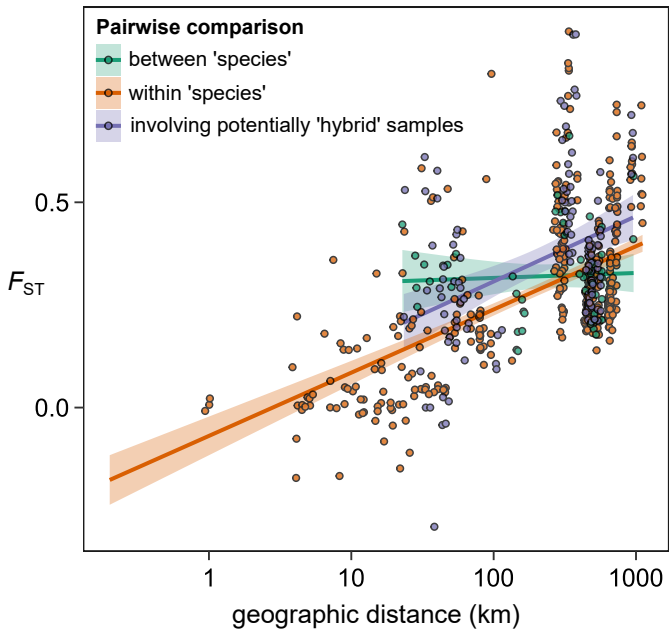

Supplement: S3 Fig — Plot of FST vs. great circle geographic distances (in log scale) between all pairs of locations in the complete dataset. Thick lines represent linear regressions and shaded areas their 95% CIs. The regression for between ‘species’ comparisons is nearly flat, while a pattern of isolation by distance is obvious in the within ‘species’ comparisons. Interestingly, comparisons involving the four samples in the potential ‘hybrid’ zone (BCH, TVN, LAP, CAN, see S2 Fig) show a similar trend of increasing genetic differentiation with increasing geographic distance but with a raised intercept, consistent with their mixed genetic makeup as evidenced in CA and Structure analysis. (PDF) [file pone.0321742.s003.pdf]
