# Supplementary material for: Genetic data suggest gene flow within a narrow hybrid zone between two recently separated species in the genus Parnassius (Lepidoptera: Papilionidae)
Source: PLoS One. 2025 Apr 24;20(4):e0321742. doi: 10.1371/journal.pone.0321742 (PMC12021245; doi:10.1371/journal.pone.0321742)
Supplement: S1 Table — (DOCX) [file pone.0321742.s004.docx]

**S1 Table. Samples employed in this study (*n* = number of genotyped individuals)**

| **code** | **site** | **country** | **lat** | **lon** | **collector** | **year** | ***n*** |
| --- | --- | --- | --- | --- | --- | --- | --- |
| TAR | Tarvisio | ITA | 46.488 | 13.617 | Valerio Sbordoni | 2003 | 28 |
| GAR | Val di Gares | ITA | 46.312 | 11.882 | Valerio Sbordoni | 2003 | 17 |
| CAN | Cansiglio | ITA | 46.069 | 12.412 | Valerio Sbordoni | 2003 | 7 |
| TVN | Val Tovanella | ITA | 46.3 | 12.28 | Sönke Hardersen | 2006 | 3 |
| BCH | Rifugio Bianchet | ITA | 46.247 | 12.171 | M. Dalla Rosa | 2007 | 1 |
| CNZ | Val Canzoi | ITA | 46.11 | 11.934 | Paolo Gratton | 2006 | 23 |
| SRV | Servo | ITA | 46.062 | 11.791 | Paolo Gratton | 2006 | 2 |
| CAU | Caupo | ITA | 45.994 | 11.834 | Paolo Gratton | 2006 | 2 |
| AUN | Croce d'Aune | ITA | 46.061 | 11.841 | Paolo Gratton | 2006 | 8 |
| GRO | Monte Grappa Ovest | ITA | 45.865 | 11.765 | Paolo Gratton | 2006 | 12 |
| LAZ | Lazzaretti | ITA | 45.92 | 11.642 | Paolo Gratton | 2005 | 21 |
| CAT | Castello Tesino | ITA | 46.067 | 11.652 | Paolo Gratton | 2005 | 16 |
| A01 | Tatz | CH | 46.326 | 7.787 | Anna Bruzinski | 2007 | 10 |
| A03 | Eison | CH | 46.151 | 7.471 | Anna Bruzinski | 2007 | 17 |
| A07 | Eison | CH | 46.401 | 7.774 | Anna Bruzinski | 2007 | 11 |
| A08 | Gental | CH | 46.728 | 8.271 | Anna Bruzinski | 2007 | 19 |
| A09 | Rosswald | CH | 46.312 | 8.043 | Anna Bruzinski | 2007 | 19 |
| A13 | Baltschiedertal | CH | 46.334 | 7.879 | Anna Bruzinski | 2007 | 12 |
| A15 | Gadmertal | CH | 46.748 | 8.383 | Anna Bruzinski | 2007 | 4 |
| SIR | Prati del Sirente | ITA | 42.174 | 13.599 | Paolo Gratton | 2003 | 31 |
| FIO | Valle Fioio | ITA | 41.962 | 13.26 | Paolo Gratton | 2003 | 26 |
| AUT | Monte Autore | ITA | 41.956 | 13.206 | Paolo Gratton | 2003 | 53 |
| CMP | Campaegli | ITA | 41.971 | 13.113 | Paolo Gratton | 2003 | 30 |
| LIV | Monte Livata | ITA | 41.933 | 13.153 | Paolo Gratton | 2003 | 47 |
| PCM | Prati di Campoli | ITA | 41.783 | 13.497 | Paolo Gratton | 2003 | 27 |
| VIG | Monte Viglio | ITA | 41.905 | 13.378 | Paolo Gratton | 2003 | 25 |
| MRS | Monte Rusco | ITA | 41.315 | 13.643 | Paolo Gratton | 2003 | 43 |
| NEB | Portella di Calcaudera | ITA | 37.925 | 14.67 | Paolo Gratton | 2003 | 26 |
